# Antibody Profiles According to Mild or Severe SARS-CoV-2 Infection, Atlanta, Georgia, USA, 2020

**DOI:** 10.3201/eid2612.203334

**Published:** 2020-12

**Authors:** William T. Hu, J. Christina Howell, Tugba Ozturk, Karima Benameur, Leda C. Bassit, Richard Ramonell, Kevin S. Cashman, Shama Pirmohammed, John D. Roback, Vincent C. Marconi, Irene Yang, Valerie V. Mac, Daniel Smith, Ignacio Sanz, Whitney Wharton, F. Eun-Hyung Lee, Raymond F. Schinazi

**Affiliations:** Emory University, Atlanta, Georgia, USA

**Keywords:** 2019 novel coronavirus disease, coronavirus disease, COVID-19, severe acute respiratory syndrome coronavirus 2, SARS-CoV-2, viruses, respiratory infections, zoonoses, antibody profiles, infection severity, immunoglobulin, neutralization, Atlanta, Georgia, USA

## Abstract

Among patients with coronavirus disease (COVID-19), IgM levels increased early after symptom onset for those with mild and severe disease, but IgG levels increased early only in those with severe disease. A similar pattern was observed in a separate serosurveillance cohort. Mild COVID-19 should be investigated separately from severe COVID-19.

Coronavirus disease (COVID-19) emerged in December 2019 (*1*,*2*), and by June 2020, »10 million persons worldwide had acquired the disease. The confirmatory test for severe acute respiratory syndrome virus 2 (SARS-CoV-2) infection remains real-time reverse transcription PCR, but this test poses challenges in terms of sensitivity (*3*), reagent or equipment availability, and specialized personnel training. Serologic assays can be readily performed in most clinical laboratories, with faster turnaround times, but their association with COVID-19 has largely been reported for hospitalized patients with severe disease (*4*; E. Adams et al., unpub. data, https://www.medrxiv.org/content/10.1101/2020.04.15.20066407v1.full.pdf). Whether mild and severe COVID-19 represent 2 interlinked stages on a severity continuum or 2 distinct phenotypes of an infectious process (*5*) remains incompletely understood; detailed cross-sectional characterization of IgM and IgG reactive against SARS-CoV-2 antigens may provide insight into the temporal evolution of antibodies. Detection of cross-reactive antibodies from a pre-2020 cohort can also indicate whether past exposure to other coronaviruses is associated with cross-reactive protection against SARS-CoV-2. 

In addition to IgG targeting the receptor-binding domain (RBD) of the spike protein subunit S1 (*6*), we developed and validated an IgM assay targeting the full-length S1 protein. We further developed and validated an IgM assay targeting the small full-length envelope (E) protein, which is highly shared between SARS-CoV and SARS-CoV-2 (*2*), is accessible on the surface, and increases during virus replication (*7*). Using these assays, we characterized the IgM and IgG profiles of participants with COVID-19, pre-2020 control participants, and a community cohort of 116 persons who had recovered from self-limited illness during March and April 2020 in Atlanta, Georgia, USA.

## The Study

We recruited 28 participants hospitalized for severe COVID-19 (20 requiring artificial ventilation; samples collected during hospitalization a median of 15.5 days after symptom onset) and 15 participants who had recently recovered from mild COVID-19 (samples collected a median of 15 days after symptom onset; Table 1). Compared with hospitalized participants, participants with mild illness were less likely to be African American (*8*) and more likely to be younger and to have nasal congestion or anosmia.

**Table 1:** Demographic and other information for persons with known coronavirus disease, pre-2020 controls, and persons with influenza-like illness but negative for SARS-CoV-2, Atlanta, Georgia, USA, 2020*

Characteristic	Hospitalized, n = 28	Mild disease, n = 15	Pre-2020 control, n = 103	p value
Sex, %				0.273
F	14 (50)	7 (47)	65 (63)	
M	14 (50)	8 (53)	38 (37)	
Median age, y (range)	61.5 (29–85)†	32 (26–81)†	62.5 (24–87)	<0.0001
Race, no. (%)				<0.0001
Asian	3 (11)	0	2 (2)	
African American	18 (64)†	1 (7)†	15 (14)	
Non-Hispanic Caucasian	6 (21)	12 (80)	82 (80)	
Hispanic	1 (4)	1 (7)	0	
Other	0	1 (7)	4 (4)	
Clinical features				
Inpatient/outpatient	28/0	1/14	NA	<0.0001
Respiratory failure requiring intubation, %	20 (71)†	0†	NA	<0.0001
Median days since symptom onset (range)	15.5 (4–42)	15 (9–33)	NA	0.427
Clinical signs/symptoms				
Cough	22 (79)	10 (67)	NA	0.473
Fever/chills	22 (79)	9 (64)	NA	0.287
Shortness of breath	20 (71)	5 (33)	NA	0.024
Myalgia	7 (25)	9 (60)	NA	0.045
Headaches	7 (25)	7 (47)	NA	0.184
Sore throat	5 (18)	6 (40)	NA	0.150
Nasal congestion/rhinorrhea	2 (7)	8 (53)	NA	0.001
Diarrhea	5 (18)	3 (20)	NA	1.000
Anosmia	1 (4)	7 (47)	NA	0.001
Fatigue	3 (11)	3 (20)	NA	0.647
Vomiting	0	1 (7)	NA	0.349
Never symptomatic	0	0	NA	0.012
Laboratory features				
SARS-CoV-2 detected by rRT-PCR	28/28	10/10	NA	<0.0001
Mean anti-S1-RBD IgG (± SD), OD	1.72 (0.72)†	0.71 (0.60)†	0.26 (0.36)	<0.0001
Mean anti-S1 IgM (± SD), OD	1.76 (0.74)	2.12 (0.53)	1.21 (0.48)	<0.0001
Mean anti-E IgM (± SD), OD	1.85 (0.90)	2.16 (0.72)	1.48 (0.71)	0.001

*E, envelope protein; NA, not applicable; OD, optical density, RBD, receptor-binding domain; rRT-PCR, real-time reverse transcription PCR; SARS-CoV-2, severe acute respiratory syndrome coronavirus 2; S1, spike protein subunit S1. †Different between patients who were hospitalized and who had mild disease at p<0.005.

**Figure 1:**
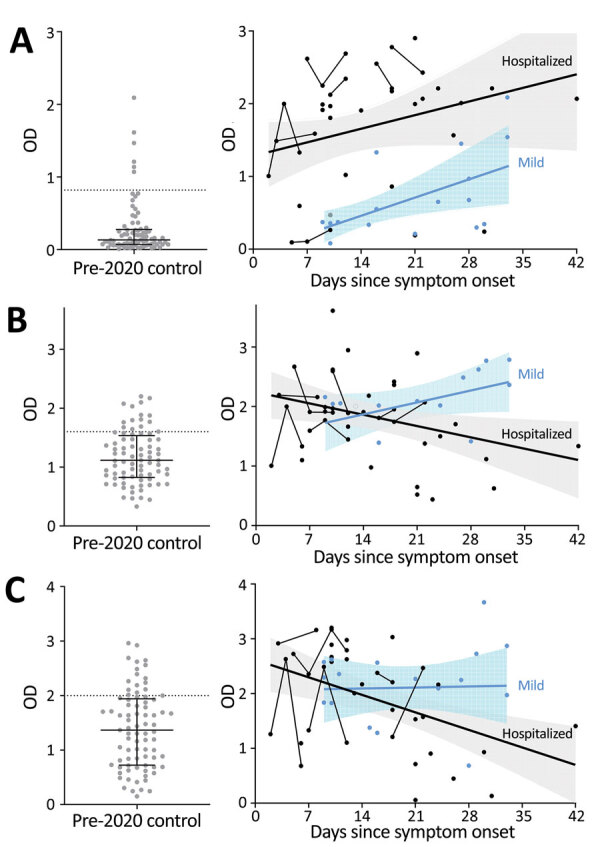
Serologic assay results for study participants with coronavirus disease (COVID-19), Atlanta, Georgia, USA, 2020. Levels of IgG against the receptor-binding domain (RBD) of the spike protein subunit S1 (A), IgM against S1 (B), and IgM against envelope protein (C) were analyzed for hospitalized patients with severe COVID-19 (black circles) and patients who had recovered from mild COVID-19 (blue circles) according to time from symptom onset. Levels in pre-2020 HC participants (gray circles) are shown for comparison; dotted lines represent optimal threshold levels for receiver operating characteristic curve analysis. Best fit lines for relationships between time since symptom onset and antibody levels were calculated separately for hospitalized participants and participants with mild COVID-19. OD, optical density.

Compared with control participants, hospitalized participants had higher levels of IgG against S1-RBD (log_10_ transformed because of nonnormal distribution; Student *t* [56.7] = 12.183; p<0.0001; Figure 1, panel A), IgM against S1 (Student *t* [33.29] = 3.713; p<0.001; Figure 1, panel B), and IgM against E (*t* [129] = 2.279; p = 0.024; Figure 1, panel C). The same was true among participants with mild illness for IgG against S1-RBD (Student *t* [116] = 4.246; p<0.0001; Figure 1, panel A), IgM against S1 (Student *t* [116] = 6.764; p<0.0001; Figure 1, panel B), and IgM against E (Student *t* [116] = 3.398; p = 0.001; Figure 1, panel C). However, an IgG diagnostic threshold of 0.82 optical density (OD) (Appendix) from the hospitalized participants identified only 4 (26.7%) of 15 participants with mild disease because of the lower IgG levels early after symptom onset in the group with mild disease. Elevated IgG only weeks after symptom onset among participants with mild COVID-19 is consistent with prior reports (*9*; E. Adams et al., unpub. data, https://www.medrxiv.org/content/10.1101/2020.04.15.20066407v1.full.pdf), and linear regression analysis projected that their IgG would reach the threshold of hospitalized participants an average of 29 days after symptom onset.

Conversely, IgM negatively correlated with time since symptom onset for hospitalized participants but not for those with mild disease. An anti-S1 IgM level of 1.60 OD from hospitalized patients during the first 21 days—before significant IgM decline—and 50-fold randomly selected control participants showed sensitivity of 81.0% and median specificity of 80.4% (range 76%–85.5%). The threshold of 1.60 OD was in range with values derived from pre-adsorption experiments that used S1 antigen (1.75 OD; Appendix) and identified participants with mild disease with sensitivity of 80.0% and median specificity of 80.5% (range 80%–86.7%). Anti-E IgM levels showed similar associations with time from symptom onset and severity but did not increase identification of COVID-19 participants.

Because many persons with mild influenza-like (ILI) symptoms in the metropolitan Atlanta area did not or could not access SARS-CoV-2 testing during early 2020, we also analyzed antibody levels in 116 adults who had recovered from self-limited ILI symptoms (Table 2). Compared with participants with mild COVID-19, this cohort was less likely to have anosmia (11% vs. 47%; p = 0.002) or fatigue (4% vs. 20%; p = 0.048) but was otherwise similar in terms of sex, race, age, and signs/symptoms. Of 31 participants with symptom onset 7–29 days before blood collection, 1 (3%) had elevated IgG, and 11 (12.9%) of 85 with symptom onset 30–60 days before participation had elevated IgG. None of the clinical signs/symptoms strongly predicted antibody levels. A liberal threshold of anti-S1 IgM >1.60 OD identified 18/31 (58%) and 57/85 (67%) participants, and a more stringent threshold of 2.00 OD to reduce false positives identified 7/31(22%) and 41/85 (48%) participants.

**Table 2:** Demographic and other information for a prospective cohort who recovered from an influenza-like illness, Atlanta, Georgia, USA, 2020

Characteristic	IgG <0.82, IgM <2.00, n = 60	IgG <0.82, IgM ≥2.00, n = 44	IgG ≥0.82, IgM <2.00, n = 8	IgG ≥0.82, IgM ≥2.00, n = 4	p value
Symptom onset, no. (%)					0.029
7–29 d earlier	23 (38)	7 (16)	1 (12)	0	
30–60 d earlier	37 (62)	37 (84)	7 (88)	4 (100)	
Sex, no. (%)					0.042
F	29 (48)	33 (75)	4 (50)	3 (75)	
M	31 (52)	11 (25)	4 (50)	1 (25)	
Median age, y (range)	45.5 (19.4–73.7)	34.9 (25.9–73.3)	43.6 (31.7–62.3)	37.3 (33.5–48.2)	0.113
Non-Hispanic Caucasian	47 (78)	36 (82)	6 (75)	4 (100)	0.715
Healthcare worker	35 (58)	27 (61)	4 (50)	2 (50)	0.918
Potential exposure to coronavirus disease	37 (62)	22 (50)	5 (62)	2 (50)	0.662
Never smoker	51 (85)	38 (86)	7 (88)	2 (50)	0.119
Clinical signs/symptoms					
Cough	38 (63)	37 (84)	2 (25)	4 (100)	0.002
Fever/chills	21 (35)	21 (48)	2 (25)	3 (75)	0.214
Shortness of breath	21 (35)	13 (29)	1 (12)	3 (75)	0.166
Myalgia	34 (57)	21 (48)	4 (50)	4 (100)	0.228
Headaches	38 (63)	21 (48)	3 (37)	3 (75)	0.238
Sore throat	27 (45)	25 (57)	3 (37)	4 (100)	0.117
Nasal congestion/rhinorrhea	37 (62)	30 (68)	3 (37)	0	0.029
Diarrhea	11 (18)	13 (29)	2 (25)	2 (50)	0.352
Anosmia	6 (10)	5 (11)	0	2 (50)	0.067
Fatigue	1 (2)	4 (9)	0	0	0.262
Vomiting	2 (3)	3 (7)	0	0	0.717

**Figure 2:**
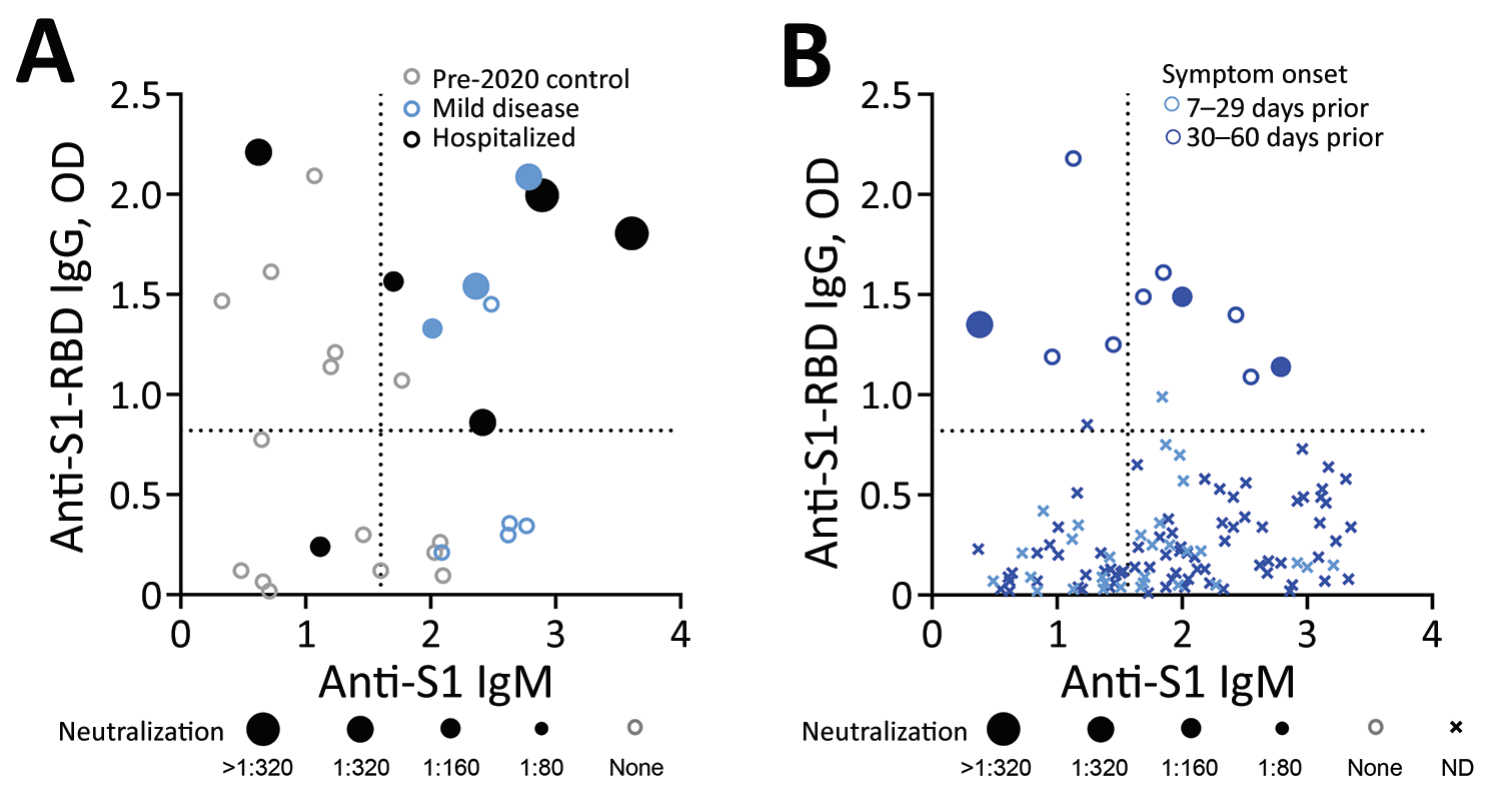
Severe acute respiratory syndrome coronavirus 2 virus neutralization measures according to anti-S1-RBD IgG and anti-S1 IgM levels, Atlanta, Georgia, USA, 2020. Open circles represent negative plaque-reduction neutralization test (PRNT) result, and solid circles represent positive PRNT result (sizes of filled circles are proportional to maximal dilution with positive PRNT result). Dotted lines indicate threshold values. A) Among participants with coronavirus disease (COVID-19) (mild disease and hospitalized), pre-2020 controls with elevated antibody levels, and pre-2020 controls with normal antibody levels, positive PRNT results were most associated with simultaneously elevated IgM and IgG levels (Appendix). B) Analysis of a group of 116 persons who reported recovery from self-limited illness 7–60 days prior showed a similar trend. ND, not done; RBD, receptor-binding domain; S1, spike protein subunit 1.

Last, we performed plaque-reduction neutralization assays (PRNT; Appendix) for a subgroup of participants with confirmed or probable COVID-19 and pre-2020 control participants (75% with elevated antibody levels; Figure 2, panel A). All 6 hospitalized participants and 5 participants with mild disease (2 weak neutralizing results <1:40) demonstrated >90% plaque reduction in Vero cells compared with 2 of 15 control participants who also showed weak neutralization. Using positive PRNT at >1:40 as a specific threshold, we found simultaneously elevated IgM and IgG most predictive of positive PRNT (p = 0.008 compared with IgM alone, p = 0.07 compared with IgG alone; Appendix), although plasma from 1 hospitalized participant with neutralizing plasma had reference IgM and IgG levels. PRNT for community participants with the 10 most elevated IgG levels showed a similar trend (Figure 2, panel B).

## Conclusions

IgM reactive toward S1 and E proteins increased early regardless of disease severity, but IgG increased early only in hospitalized participants with severe COVID-19. This pattern was observed in a separate cohort of community participants who had recovered from self-limited ILI. Positive PRNT—a surrogate for antibody-mediated immune protection—may be better associated with elevated IgM and IgG than either antibody alone. 

A diagnostic algorithm of IgG from hospitalized participants performed poorly for detection of mild COVID-19. Similarly, other studies found delayed or low-to-medium neutralizing antibody titers in persons who recovered from mild COVID-19 (E. Adams et al., unpub. data, https://www.medrxiv.org/content/10.1101/2020.04.15.20066407v1.full.pdf; F. Wu et al., unpub. data, https://www.medrxiv.org/content/10.1101/2020.03.30.20047365v2). The delayed increase in IgG and neutralizing antibodies in persons with mild COVID-19 also suggests that mild cases do not necessarily represent an intermediate stage between severe and asymptomatic COVID-19. A corollary of slow IgG increases in persons with mild COVID-19 may be longer persistence of IgM, but more definitive characterization of IgM+ memory B cells (*10*) and long-term decay of antibody levels (*11*) is needed.

Our study has limitations. Our small cross-sectional cohort of patients with well-characterized and laboratory-confirmed COVID-19 limits generalization. The overrepresentation of African Americans in the more severely ill cohort may mediate some differences in antibody profiles (*8*), and we did not measure IgA levels or antibodies targeting other SARS-CoV-2 gene products (currently under development and validation). We also did not measure antibody levels in historic SARS or MERS case-patients, and cross-reactive antibody response against homologous regions cannot be ruled out. 

We did confirm a complex relationship between antibody levels, disease severity, and time since symptom onset. Examining IgM and IgG against multiple SARS-CoV-2–related antigens may thus better inform natural history and vaccine studies than any one antibody.

Appendix Supplemental methods and results for study of antibody profiles according to mild or severe SARS-CoV-2 infection, Atlanta, Georgia, USA, 2020.
