# Supplementary material for: Antibody Profiles According to Mild or Severe SARS-CoV-2 Infection, Atlanta, Georgia, USA, 2020
Source: Emerg Infect Dis. 2020 Dec;26(12):2974–8. doi: 10.3201/eid2612.203334 (PMC7706962; doi:10.3201/eid2612.203334)
Supplement: Appendix — Supplemental methods and results for study of antibody profiles according to mild or severe SARS-CoV-2 infection, Atlanta, Georgia, USA, 2020. [file 20-3334-Techapp-s1.pdf]

Article DOI: https://doi.org/10.3201/eid2612.203334

Antibody Profiles According to Mild or Severe SARS-CoV-2 Infection, Atlanta, Georgia, USA, 2020

Appendix

Materials and Methods

Standard Protocol Approvals, Registrations, and Patient Consents

This study was approved by Emory University Institutional Review Board. Written consents were obtained from all participants or their legally authorized representatives (when appropriate). Sample size was calculated based on one previous study (*1*) when the current study began using a more conservative effect size (0.8 vs. >1), with an estimated disease prevalence of 5%-20%. Plasma was collected from 15 hospitalized participants, ten mild participants, and all pre-2020 HC and those with negative rRT-PCR, while serum was collected from the remaining 13 hospitalized participants and five mild participants.

Study Participants

All hospitalized participants had rRT-PCR confirmation. Mild participants were recruited up until 35 days (inclusive) following symptom onset. Among the mild participants, ten had rRT-PCR confirmation, four developed influenza-like illness (ILI) following direct contact with confirmed COVID-19 cases but were not eligible for rRT-PCR, and one developed ILI following direct contact with confirmed COVID-19 cases but did not seek rRT-PCR. Mild participants did not differ in antibody levels or time since symptom onset according to rRT-PCR result availability, and excluding them from the cross-sectional analysis of antibody level vs. time did not change the time-associated trends. Furthermore, two of four without rRT-PCR confirmation (vs. one of four with rRT-PCR confirmation) had positive neutralizing antibodies (see below and results). Therefore, the five participants without rRT-PCR confirmation were thus included in analysis. Pre-2020 HC participants (n=103) were recruited through inflammation studies targeting the young (PI: WTH),(*2*) middle-aged (PI: WW),(*3*) or older (PI: WTH) healthy adults.

A large cohort of people who recovered from self-limited ILI but did not seek rRT-PCR confirmation were prospectively recruited as part of a larger baseline serological surveillance study conducted at Emory University during April and May 2020. Participants were screened for temperature and COVID-19-related symptoms to ensure they were asymptomatic at time of blood collection, and asked to recall if they had a standardized list of any influenza- or COVID-19-like symptoms in the prior four months. Dates of symptom onset and resolution were also recorded, along with information on if they were healthcare workers and if they were exposed to someone with confirmed COVID-19. Participants who recorded any symptoms as well as onset within the past 7-60 days (116/369, 31%) were included in the current study.

Specimen Collection

For subjects with plasma (n=244), blood was collected in K_2EDTA tubes and placed on ice immediately. Blood samples were centrifuged at 4^oC and 2500 g for 10 minutes, and plasma was removed, aliquoted, labeled, and frozen immediately at -80^oC until analysis. For subjects with serum (n=28), blood was collected in silicone-coated tubes and allowed to clot in the upright position at room temperature for 30 minutes. After centrifugation at 4^oC and 2500 g for 10 minutes, serum was removed, aliquoted, labeled, and frozen immediately at -80^oC until analysis

IgG Serological Assay

A commercial anti-S1 receptor binding domain (RBD) IgG indirect ELISA assay (GenScript, Piscataway, NJ) was purchased and performed per manufacturer's protocol, except two plasma dilutions (1:16 and 1:64) were selected from a range of 1:8 – 1:256 performed in a subgroup of COVID-19 and pre-2020 HC participants. To derive an empirical threshold for COVID-19, receiver operating characteristics (ROC) curve analysis using 18 hospitalized COVID-19 participants (all with rRT-PCR confirmation) and 75 pre-2020 HC participant showed area under the curve (AUC) of 0.941 (95% CI 0.891-0.990). None of the pre-2020 HC participants were tested for IgG against SARS-CoV or other coronaviruses.

Development and Validation of IgM Serological Assays

To detect IgM targeting S1 and E proteins, we developed two novel assays based on our prior experience in modifying ELISA and multiplex immunoassays to measure targets of low abundance. Synthetic SARS-CoV-2 S1 (230-01101-100, produced from *E. coli*) and SARS-

related E (228-11400-2, produced from *E. coli*) peptides were purchased from RayBiotech (Peachtree Corners, GA). 100 μL of 2.5 μg/mL antigen in PBS with 2% non-fat dried milk (nfdm) and 0.1% Tween was applied to standard 96-well plate at 4ºC overnight. Alternate diluents (including sodium bicarbonate buffer, TBS, PBS with 1% nfdm, PBS with 2% albumin) and concentrations (1 μg/mL, 4 μμ/mL) resulted in insufficient sensitivity or greater non-specific binding. During assay development, multiple plasma dilutions (1:2, 1:8, 1:32…1:1,024), blocking conditions (PBS with 0.1%, 1%, 5% albumin, 8% casein, or 4% nfdm, or PBS with 4% nfdm and 0.1% Tween), and detecting antibody mix (donkey- vs. goat-derived; PBS, PBS with 1-3% nfdm with or without Tween) were tested to derive the final protocol. Following overnight coating, ELISA plates were washed three times with PBS and blocked with PBS with 4% nfdm before 50 μL of 1:20,000 affinity-purified goat anti-human IgM fc (109-035-043, Jackson ImmunoResearch Laboratories, West Grove, PA; in PBS) was added to each dilution condition for 30 min. Wells were then washed again, treated with strepavidin-HRP (1:200, 50 μL per well) for 20 min in the dark, washed, incubated with substrate mix for 20 min in the dark, and treated with reaction stop solution. Plates were then read at 450 nm (Molecular Devices, SpectraMax-M2) followed by background (570 nm) subtraction to derive relative units (in optical density [O.D.]). We did not include an IgG-removal (from plasma) process because the affinity-purified anti-human IgM Fc antibody does not react to human IgG in our hands (using humanized monoclonal antibody standards).

To demonstrate assay specificity in the finalized protocol, we pre-adsorbed plasma from RT-PCR confirmed COVID-19 and pre-2020 HC subjects using soluble S1 or E protein (corresponding to each assay). In pre-adsorption experiments, antigen-specific antibodies present in the plasma would be bound to soluble antigens of markedly greater concentration, and remaining antibodies which bind to the plated antigens then bind either 1) non-specifically, 2) preferentially to post-translationally modified native protein, or 3) to antigen in a non-soluble conformational state. For each tested sample, we calculated O.D. difference between O.D. with and without antigen pre-adsorption (Appendix Figure 1). This showed linear relationships between antibody levels associated with plated antigen (X-axis) and soluble antigen (Y-axis) when the former exceeds 1.75 O.D. in S1 (R^2=0.789) and 2.01 O.D. (R^2=0.443) in E. Because antigen (S1 or E) binding to ELISA plates requires a hydrophobic reaction between *synthetic protein* and the plastic surface (sometimes facilitated by the use of sodium bicarbonate coating

buffer) which may alter the target antigen's conformation, we do not consider detected antibody levels below these two thresholds as *exclusively* non-specific. For the purpose of this descriptive study, we therefore elected to report the total measured antibody levels. We did not have access to stored blood samples from patients with SARS-CoV, MERS, or other coronavirus infections to determine if they have elevated IgM levels beyond the empirically determined thresholds. While IgM levels normalized within weeks to months after infection in the 2003 SARS outbreak (*4,5*) and no pre-2020 participant from our studies had reported prior MERS infection, we do not exclude the possibility that even low degree homology between SARS-CoV-2 and other coronaviruses could have contributed to the artificially high O.D. from pre-2020 HC participants.

Viral Neutralization Assays

Viral neutralization was determined by plaque reduction neutralization test (PRNT) using enzyme-linked immunospot as a read out. Serially diluted (1:20 – 1:2560) plasma was incubated with SARS-CoV-2 USA-WA1 (≈70 foci/well) for 1 hour at 37°C, along with negative and positive (convalescent serum, BEI Resources, Manassas, VA) control samples. Confluent Vero cells were subsequently incubated with the virus-plasma mix for 1 hr, followed by addition of overlay media (Opti-MEM, 2% FBS, 2.5 μg/mL amphotericin B, 20 μg/mL Ciprofloxacin, 2% methylcellulose) and incubation for three days. PRNT was then performed using monoclonal anti-SARS recombinant IgG1 (NR-52392, BEI) as a positive control, with >90% reduction considered as a positive response in this study. All assays were performed by experienced scientists blinded to diagnosis.

Statistical Analyses

All statistical analyses were performed using SPSS 26 (IBM SPSS, Armonk, NY) except curve-fitting was performed in Prism 8.4.3 (GraphPad Software, San Diego, CA). Chi-squared or Fisher's exact test was used to analyze differences in categorical variables and Student's T-tests were used to analyze differences in continuous variables between hospitalized and mild COVID-19 participants. Only non-HC participants were analyzed for differences in clinical symptoms. Anti-S1-RBD IgG levels were $\log_{10}$-transformed before analysis due to its non-normal distribution. Given the expected effect sizes, Bonferroni correction was used to adjust for multiple comparisons.

For each antibody, linear regression between days after symptom onset and antibody levels was compared against other higher order models (second- or third-order polynomial, and exponential growth for anti-S1-SBD IgG in recovered cases) based on Akaike Information Criteria. Linear functions provided better fit than more complex models for all three antibodies.

Receiver-operating characteristic (ROC) curve analysis for IgM was trained on hospitalized COVID-19 participants up to 21 days after symptom onset and 75 randomly selected pre-2020 HC participants 50 times to derive antibody thresholds. These thresholds were then tested in mild COVID-19 participants and the remaining pre-2020 HC participants to report median sensitivity and specificity. One example is shown in Appendix Figure 2.

To determine the best predictors of positive PRNT results, we analyze elevated anti-S1 IgM, elevated anti-S1-RBD IgG, and elevated levels of both relative to PRNT outcomes in eight participants with mild COVID-19, six participants hospitalized for severe COVID-19, and 16 pre-2020 HC participants (11 with elevated anti-S1 IgM or anti-S1-RBD IgG). Sensitivity and specificity improvement was determined by McNemar's χ^2 Test using positive (for sensitivity) or negative (specificity) PRNT cases (Appendix Table) (*6*).

References

1. Amanat F, Nguyen THO, Chromikova V, Strohmeier S, Stadlbauer D, Javier A, et al. A serological assay to detect SARS-CoV-2 seroconversion in humans. Nat Med 2020;26:1033–6. https://doi.org/10.1038/s41591-020-0913-5

2. Hu WT, Howell JC, Ozturk T, Gangishetti U, Kollhoff AL, Hatcher-Martin JM, et al. CSF cytokines in aging, multiple sclerosis, and dementia. Front Immunol. 2019;10:480. PubMed https://doi.org/10.3389/fimmu.2019.00480

3. Wharton W, Kollhoff AL, Gangishetti U, Verble DD, Upadhya S, Zetterberg H, et al. Interleukin 9 alterations linked to Alzheimer disease in African Americans. Ann Neurol. 2019;86:407–18. PubMed https://doi.org/10.1002/ana.25543

4. Chang SC, Wang JT, Huang LM, Chen YC, Fang CT, Sheng WH, et al. Longitudinal analysis of severe acute respiratory syndrome (SARS) coronavirus-specific antibody in SARS patients. Clin Diagn Lab Immunol. 2005;12:1455–7. PubMed https://doi.org/10.1128/CDLI.12.12.1455-1457.2005

5. He Z, Dong Q, Zhuang H, Song S, Peng G, Luo G, et al. Kinetics of severe acute respiratory syndrome (SARS) coronavirus-specific antibodies in 271 laboratory-confirmed cases of SARS. Clin Diagn Lab Immunol. 2004;11:792–4. PubMed https://doi.org/10.1128/CDLI.11.4.792-794.2004

6. Trajman A, Luiz RR. McNemar χ^2 test revisited: comparing sensitivity and specificity of diagnostic examinations. Scand J Clin Lab Invest. 2008;68:77–80. PubMed https://doi.org/10.1080/00365510701666031

Appendix Table. Relationship between anti-S1 IgM, anti-S1-RBD IgG, and PRNT results in mild and hospitalized COVID-19 participants (greater than 90% plaque reduction at >1:40 dilution was considered a true positive response)

Variable	Elevated anti-S1 IgM (> 1.60 O.D.)	Elevated Anti-S1-RBD-IgG (> 0.82 O.D.)	Elevated anti-S1 IgM & anti-S1-RBD IgG
(+)PRNT	7/9	7/9	7/9
(-) PRNT	11/21	7/21	2/21
Sensitivity for (+)PRNT	77.8%	77.8%	77.8%
Specificity for (+)PRNT	47.6%	66.7%	90.5%*
Accuracy for PRNT	56.7%	70.0%	86.7%

*p=0.008 compared to IgM only and p=0.07 compared to IgG only by McNemar's χ^2 test.

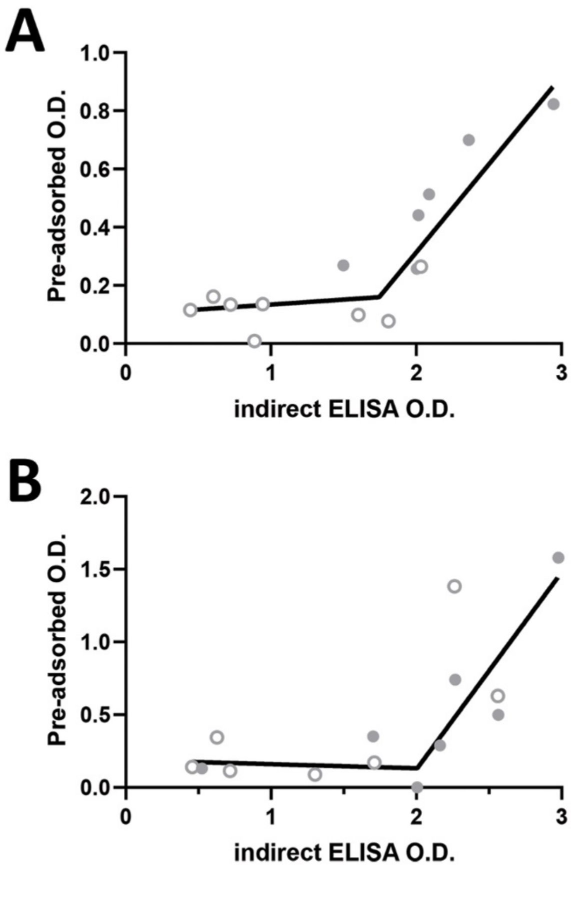

Appendix Figure 1. Correlation between directly measured antibody levels and calculated pre-adsorbed antibody levels using soluble S1 and E proteins.

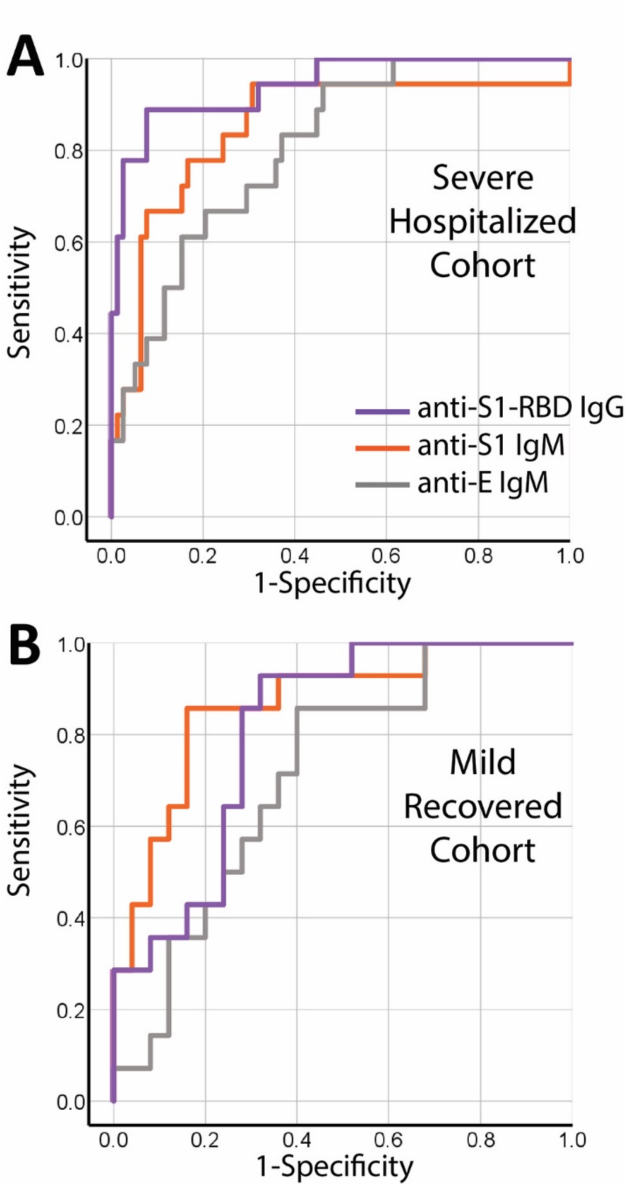

Appendix Figure 2. Example of ROC curve analysis using the three serological assays to distinguish severe hospitalized (A) and mild recovered (B) COVID-19 participants from randomly selected HC participants. This process was repeated 50 times to generate median O.D. for anti-S1 IgM and specificity.
